# Inflammatory discoveries two years after acute severe COVID-19: a longitudinal biomarker profile assessment in long COVID individuals in the Brazilian Amazon

**DOI:** 10.3389/fimmu.2024.1520193

**Published:** 2024-12-23

**Authors:** Alex Bezerra Silva Maciel, Arlene Santos Pinto, Bernardo Maia Silva, Cassia Luz Goulart, Luis Felipe Alho Silva, Amanda Silva Chaves, Gabriel Santos Mouta, Camila Miriam Suemi Sato, Jefferson Valente, Victor Irungu Mwangi, Gisely Cardoso de Melo, Wuelton Monteiro, Marcus Lacerda, Guilherme Peixoto Tinoco Arêas, Vanderson Souza Sampaio, Allyson Guimaraes Costa, Fernando Almeida-Val

**Affiliations:** ^1^ Diretoria de Ensino e Pesquisa, Fundação de Medicina Tropical Dr Heitor Vieira Dourado, Manaus, AM, Brazil; ^2^ Programa de Pós-Graduação em Medicina Tropical, Universidade do Estado Do Amazonas, Manaus, AM, Brazil; ^3^ Programa de Pós-graduação em Biologia da Interação Patógeno Hospedeiro, Instituto Leônidas e Maria Deane (ILMD-FIOCRUZ/AM), Manaus, AM, Brazil; ^4^ Programa de Pós-graduação em Imunologia Básica e Aplicada, Universidade Federal do Amazonas, Manaus, AM, Brazil; ^5^ Diretoria, Instituto Todos Pela Saúde, São Paulo, SP, Brazil; ^6^ Diretoria de Ensino e Pesquisa, Fundação Hospitalar de Hematologia e Hemoterapia do Amazonas, Manaus, AM, Brazil

**Keywords:** long COVID, cytokines, SARS-CoV-2, inflammation, biomarkers

## Abstract

**Background:**

In SARS-CoV-2 infection, cytokines and laboratory biomarkers play a key role in disease progression and their long-term levels have been associated with the outcome of long COVID-19.

**Objectives:**

I) study the levels of cytokines, hematological and biochemical biomarkers in the acute and post-acute phases of COVID-19 disease; and II) assess the impact of COVID-19 vaccine doses on fatigue symptoms.

**Methods:**

This study is an exploratory cohort nested within a clinical and laboratory follow-up of surviving participants after pre-vaccine acute COVID-19 infection with severe clinical manifestations. We analyzed the inflammatory biomarker profiles of fifty SARS-Cov-2 negative healthy controls from before the COVID-19 pandemic, and eighty patients in the acute phase (Day 1, Day 7 and Day 14), and during 4 months and 2 years after hospitalization.

**Results:**

Four months after hospitalization, 91.3% (73/80) of patients exhibited onset of long COVID symptoms, which persisted in 63.7% (51/80) after 2 years. Comparing the baseline values of the cytokines in the controls versus the follow-up times, the cytokines IL-6, IL-8 and IL-10 were high in the acute phase, declining over time after the individual’s recovery, while IL-1β showed an inverse variation, remaining high after 2 years. IL-1β, IL-10, and TNF increased over time post-acute infection, indicating a long-term inflammatory response. Vaccination with four doses, compared to three doses, showed a slight protective effect against fatigue symptoms in the male population (IRR 0.48, 95% CI 0.22 - 1.02; *p=0.054*). Neutrophil and leukocyte counts showed a significant reduction 2 years after hospitalization. However, platelet count was the laboratory biomarker that best reflects the prediction of long COVID symptoms up for to 2 years.

**Conclusion:**

Although the frequency of long COVID symptoms declines over time after the acute illness, symptoms continue to persist 2 years after hospital discharge. Vaccination with a fourth dose booster appears to significantly influence reduction of symptoms associated with long COVID fatigue among the males. We further identified important laboratory biomarkers for long COVID. Elevated levels of IL-1β, IL-10, and TNF, along with low levels of IL-6, IL-18, and IL-12p70, also offer new insights into the inflammatory state in long COVID.

## 1 Introduction

COVID-19 primarily affects the respiratory system; however, months or even years after recovery, it can manifest as a broad spectrum of chronic symptoms impacting various organs and tissues (1). During the acute illness there is an inflammatory response, characterized by high levels of cytokines and chemokines, such as tumor necrosis factor alpha (TNF-α), interleukin-1 beta (IL-1β), interleukin-2 (IL-2), interleukin-6 (IL-6) and interleukin-8 (IL-8), was observed in patients with COVID-19, especially in patients with a severe disease, compared to individuals without severe respiratory disease (2). This inflammatory profile in the acute phase and the development of severe disease may play a crucial role in the frequency of long COVID-19. Globally, it is estimated that at least 65 million individuals present symptoms of long COVID, with an incidence of approximately 10% among survivors (3). Risk factors for developing this condition appear to be female individuals, type 2 diabetes, presence of specific autoantibodies, lower income, inability to rest adequately after hospitalization, or other comorbidities (3). It is a multisystemic condition presenting a wide range of manifestations, including gastrointestinal disturbances, headaches, skin rashes, shortness of breath, chest pain, cognitive impairment, loss of smell and taste, profound fatigue, and musculoskeletal pain (4). Long COVID is a condition defined as the continuation or emergence of new symptoms at least three months after the initial SARS-CoV-2 infection, with symptoms persisting for a minimum of two months (5). The underlying mechanisms of long COVID remain only partially understood. Studies have identified elevated levels of inflammatory cytokines, including interleukins (IL-1β, IL-6, IL-12, IL-18, IL-33), interferons (IFN-α, IFN-γ), tumor necrosis factor (TNF), and growth factors like TGF-β, in patients with long COVID (6). The complement system is also currently considered to play an important role in the pathogenesis of long COVID. Patients with severe disease have significant shedding of C5a and C5b-9 fragments (7) and high levels of MASP-2/C1Inh and C1s/C1Inh in their plasma, indicative of activation of the lectin pathway and the classical pathway, respectively (8). In addition, fragments of C2 molecules, C5bC6/C7 complex, Factor B, von Willebrand factor (vWF) and thrombospondin-1 (TSP1) were observed in a patient with acute COVID-19 and 6 months after hospital discharge, which increases endothelial damage and inflammation in the individual’s blood vessels (9).These circulating cytokine levels, some hematologic abnormalities such as alterations in the coagulation cascade, and molecules involved in the innate and adaptive immune response have already been suggested as valuable diagnostic and prognostic biomarkers of COVID-19 (10–12). Furthermore, the persistence of these pro-inflammatory cytokines has been implicated in the maintenance of chronic inflammation and the formation of fibrinolysis-resistant micro clots, further exacerbating the condition of long COVID (13). This hypothesis is supported by findings showing elevated levels of neutrophil activation proteins and other inflammatory molecules in patients with long COVID (14). Long COVID may develop a persistent inflammatory state. Discovery this profile of Laboratorial Biomarkers and Cytokines would be essential for our understanding of the pathophysiology behind long COVID as well as provide guidance for laboratory diagnostic testing and clinical management. Based on this, we hypothesized that long COVID may be caused by abnormal, sustained, and elevated levels of biomarker inflammation present in the blood after an acute severe SARS-CoV-2 infection has subsided. Thus, to explore this hypothesis we longitudinally characterized the inflammatory profile in long COVID. Furthermore, based on recent studies on the influence of vaccination and gender on long-term symptoms, we sought to stratify and analyze vaccination status to identify any differences between individuals.

## 2 Methods

### 2.1 Study design and ethical considerations

This is a clinical and laboratory exploratory longitudinal nested cohort study of surviving COVID-19 participants with severe clinical presentation in Manaus, Amazonas, Brazil. This study consisted of a longitudinal assessment of participants up to 24 months after acute infection. This study was approved by the Brazilian Research Ethics Committee under opinion no. 52378221.7.0000.0005. All participants provided written consent before participation in the study. The collection and use of the negative control group was approved by the Ethics Committee of the Fundação Hospitalar de Hematologia e Hemoterapia do Amazonas (HEMOAM) (CAAE 56413316.9.0000.0009).

### 2.2 Study participants and data collection

We conducted a 2-year follow-up prospective cohort of a previously published clinical trial (Jeronimo CMP, et al) (15). Results from the four-month clinical follow-up have been previously published by (Barros CMSS, et al) (16). All assessments in this study were performed at the Fundação de Medicina Tropical Dr Heitor Vieira Dourado (FMT-HVD), a reference institution for infectious diseases in the northern region of the country. In this study, clinically stable individuals residing in Manaus-AM of both sexes, aged 18 years or older, who were hospitalized at the Hospital Delphina Rinaldi Abdel Aziz and Emergency Room in Manaus-AM during the first wave of COVID-19 from March to May 2020 and positive for SARS-CoV-2 by RT-qPCR at hospitalization were eligible to participate. Patients were invited by phone to participate in a structured long COVID follow-up multidisciplinary program. Moreover, 50 healthy individuals were included as a control group to compare the cytokine profile at baseline. These control samples were collected from donors at HEMOAM in Manaus, Amazonas, Brazil, before the start of the COVID-19 pandemic. The control samples were negative for SARS-CoV-2 and were only analyzed for baseline comparison. All participants were evaluated during the acute phase (during the hospitalization period), after 4 months, and 2 years after the acute disease. All participants had their data and information collected through a clinical questionnaire consisting of 34 items on self-reported signs and symptoms at the time of visits, aiming to verify the persistence of symptoms after hospitalization. Additionally, they underwent a battery of laboratory tests to evaluate the persistence of inflammatory and metabolic alterations over time. Clinical data with demographic characteristics (age, sex, preexisting comorbidities, signs and symptoms, vaccination status), laboratory test results such as complete blood count, liver function, kidney function, electrolyte tests, medical history, and clinical evolution of patients throughout the course of the disease and recovery were stored in REDCap. For the definition of the severity criteria of acute COVID-19 we used the Coronavirus Disease 2019 (COVID-19) Treatment Guidelines - National Institutes of Health classifying participants into (Moderate illness: Individuals who showed evidence of lower respiratory disease during clinical assessment or imaging and who have an oxygen saturation measured by pulse oximetry (SpO_2_) ≥94% on admission. Severe illness: Individuals who have an SpO_2_ 30 breaths/min, or lung infiltrates >50% and Critical illness: Individuals who had respiratory failure, septic shock, or multiple organ dysfunction) (17). Long COVID was defined as the presence of at least one sequela symptom after three months based on the WHO Long COVID case definition, confirmed intermittently or continuously lasting for at least 2 months with no other explanation (18).

### 2.3 Sample collection

Serum samples were collected from all patients during their hospital stay for routine laboratory tests and stored for subsequent inflammatory profiling. During the convalescent phase, approximately 50ml of peripheral blood samples were collected from participants in vacuum tubes with sodium heparin (BD Vacutainer, Becton Drive, Franklin Lakes, USA) early in the morning. After collection, the samples were transported for hematology and biochemistry tests at the Clinical Analysis Laboratory at FMT-HVD. Aliquots for the inflammatory profile study were centrifuged and stored in a -80°C freezer until analysis.

### 2.4 Cytokine and laboratorial biomarkers serum levels

The quantification of circulating cytokines in patient and controls serum samples was performed using the Cytometric Bead Array (CBA) technique with the Human Inflammatory Cytokine Kit (BD Biosciences, San Jose, USA), according to the manufacturer’s instructions. The BD™ CBA Kit uses a series of particles (beads) of known size and distinct fluorescence intensity to simultaneously detect various soluble cytokines through a capture surface. Each capture bead in the kit was conjugated with a specific antibody. Samples were run in a FACS CantoII (BD^®^ Biosciences, San Jose, CA, USA) and the FCAP Array software v3 (Soft Flow Inc., USA) was used for data analysis. The quantified cytokines were IL-1β, IL-6, IL-8, IL-10, IL12p70, and TNF, with concentrations in pg/mL and Mean Fluorescence Intensity (MFI) of each cytokine. The hematological and biochemical laboratory markers investigated were hemoglobin, hematocrit, platelets, leukocytes, neutrophils and CK, CK-MB, creatinine, urea, aspartate aminotransferase, alanine aminotransferase, total bilirubin, direct bilirubin, indirect bilirubin, sodium, potassium, ferritin, lactate dehydrogenase, glucose, triglyceride, total cholesterol and HDL.

### 2.5 Statistical analysis

Descriptive statistics (mean, standard deviation) and analytical statistics (chi-square and Wilcoxon tests) were performed as needed at a 95% significance level after initially being subjected to a Shapiro-Wilk test for normality. A mixed linear random effect model was conducted to evaluate variations in plasma levels of inflammatory markers over time and their relationship with long COVID symptoms. To understand the direct and indirect impact of vaccination on long COVID, we conducted causal mediation analyses, where the treatment was vaccination, and the outcome was the frequency of long COVID symptoms. We adjusted two log-binomial regression models by sex. The most scientifically significant inflammatory and laboratory markers were considered for subsequent network development using the open-source software Cytoscape (version 3.9.1) (Cytoscape Consortium San Diego, CA, USA). In all cases, significance was considered at p-value <*0.05*. A negative binomial Poisson regression model with a 95% confidence interval was used in the univariate and multivariate analysis with robust variance to analyze laboratory and inflammatory parameters and their effects on long COVID. Statistical analyses were performed using STATA version 16.0, GraphPad Prism version 10.0.1 (GraphPad Software, San Diego, CA, USA), and RStudio 4.0.2.

## 3 Results

### 3.1 Clinical and laboratory characteristics of study participants

During the study recruitment period, 239 surviving patients were contacted by telephone, and 159 were excluded due to eligibility criteria, refusals to participate, and loss of follow-up. A total of 80 recovered participants were included. The general characteristics of the acute phase are presented in **Table 1**. During the acute phase, female was the most predominant gender with 56.3% (45/80) of the population. The majority, 71.3% (57/80), were patients with severe infection, 18.8% (15/80) had moderate acute infection and 10% (8/80) had critical illness. 90% (72/80) had pre-existing comorbidities at admission, of which systemic arterial hypertension 33/80 (41.3%) and Diabetes Mellitus 18/80 (25%) were the most frequent comorbidities.

**Table 1:** Demographic and clinical findings of patients at baseline.

Variables	Day 1, hospital admission (N=80)
Women, n (%)	45/80 (56.3%)
Age, mean ± SD, y	52.7 ± 1.60
Race	
White, n (%)	13/80 (16.2%)
Mixed, n (%)	59/80 (73.7%)
Black, n (%)	5/80 (6.2%)
Indigenous, n (%)	3/80 (3.7%)
Symptom Onset Date until Hospital Admission, (IQR), days	14 (2-68)
Acute Phase, Severity	
Mild, n (%)	15/80 (18.8%)
Severe, n (%)	57/80 (71.3%)
Critic, n (%)	8/80 (10%)
Previous comorbidity, n (%)	72/80 (90%)
Diabetes, n (%)	18/80 (25%)
Arterial hypertension, n (%)	33/80 (41.3%)
Liver disease, n (%)	9/80 (12.5%)
Smoking, n (%)	1/80 (1.3%)
Alcoholism, n (%)	18/80 (25%)
BMI, median (IQR)	31 (17.9-52.1)

IQR, interquartile range; BMI, body mass index (calculated as weight in kilograms divided by height in meters squared).

The leukocyte median count was 9.94 mm^3^ (2.7 – 28.2) in the acute phase, a higher value compared to that after 2-years result similar to the neutrophil count. Platelets had a median value of 304,000 mm³ (8.2 – 649) in the acute phase, slightly higher than after 2 years, 224,000 mm³ (243-424) (**Table 2**).

**Table 2:** Clinical and Laboratory findings of patients in acute phase and 2 years follow-up.

Variables	Day 1, hospital admission (N=80)	Follow-Up, 2 years (N = 80)	*p-value*
Heart rate, mean ± SD, bpm	86.5 ± 1.38	77.2 ± 15.4	**<0.001**
Respiratory rate, mean ± SD, rpm	23.1 ± 0.60	19.8 ± 3.6	**0.003**
Systolic blood pressure, mean ± SD, mm/Hg	132.4 ± 2.0	138.8 ± 13.8	**0.003**
Diastolic blood pressure, mean ± SD, mm/Hg	82.1 ± 1.29	84.5 ± 11.6	0.180
Oxygen saturation, median (IQR), %	96 (81-100)	97.7 (95-100)	**0.001**
Hemoglobin, mean ± SD, g/dL	12.1± 0.1	14.5 ± 1.5	**<0.001**
Hematocrit, median (IQR), %	39.2 (20.9 – 48.2)	43.9 (4.7-55)	**<0.001**
Platelet’s count, median (IQR), mm³	304 (8.2 – 649)	224 (243-424)	**<0.001**
Leukocyte, median (IQR), mm³	9.94 (2.7 – 28.2)	7.33 (5218-14110)	**<0.001**
Neutrophil, mean ± SD, %	76.3 ± 1.5	57.4 ± 8.8	**<0.001**
CK, median (IQR), U/L	76.6 (14.8 – 8725)	107 (18-1418)	0.127
CK-MB, median (IQR), U/L	18.6 (6.5 – 49.7)	14 (6-56)	**<0.001**
Glucose, median (IQR), mg/dL	162 (98 – 370)	100 (60-361)	**<0.001**
Total bilirubin, median (IQR), mg/dL	0.4 (0.15 – 3.33)	0.57 (2-2.1)	**0.040**
Lactate dehydrogenase, median (IQR), U/L	485 (32.4 – 2046)	375 (255-771)	**0.019**
Aspartate aminotransferase, median (IQR), U/L	38.8 (9.1 – 162)	22 (10-67)	**<0.001**
Alanine aminotransferase, median (IQR), U/L	46.3 (11.3 – 285.6)	28.8 (9-145)	**<0.001**
Creatinine, median (IQR), mg/dL	0.82 (0.27 – 4)	0.9 (0.5-2.1)	0.146
Urea, median (IQR), mg/dL	29.6 (14.5 – 128)	31.5 (16-77)	0.390
Sodium, median (IQR), mEq/L	140 (131 – 152)	138 (121 – 184)	**<0.001**
Potassium, median (IQR), mEq/L	4.2 (3.2 – 5.3)	4.3 (3.2 – 6.5)	**0.030**
Ferritin, median (IQR), ng/dL	522 (40.5 – 4650)	132 (10 – 717)	**<0.001**
Total cholesterol, median (IQR), mg/dL	164.3 (99.6 – 234.6)	217 (115-359)	**0.001**
Triglycerides, median (IQR), mg/dL	186.3 (58.8 – 563.2)	185 (39-945)	0.868
HDL, median (IQR), mg/dL	39.5 (15.3 – 273)	42 (24-82)	0.252

Wilcoxon test; The significance level for all criteria is set to p < 0.05; IQR, interquartile range; CK, Creatine kinase; CKMB, Creatine kinase MB; HDL, High density lipoprotein.Significant differences of p<0.05 are represented in bold.

### 3.2 Acute and long COVID clinical presentations

**Figure 1:**
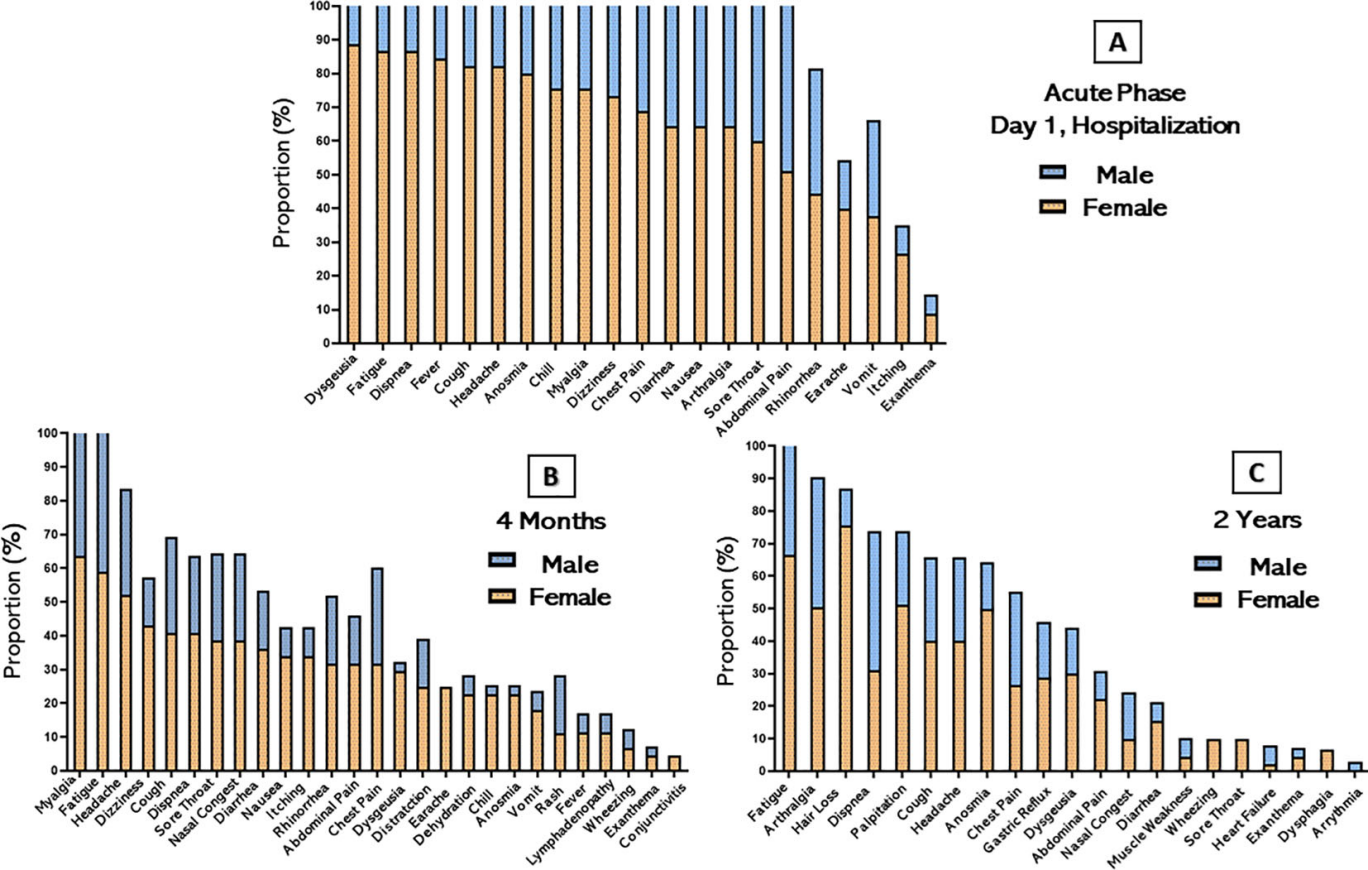
Distribution of persistent symptoms or signs after acute COVID-19 stratified by sex. The bars represent the percentage of participants in their respective categories who presented and/or continued to present each symptom over time. **(A)** Acute phase of hospitalization **(B)** 4 months after hospital discharge and **(C)** 2 years after hospital discharge.

During the acute phase of the disease, fatigue was the most frequently reported symptom upon admission by both sexes, present in all male patients and 86.6% of female patients. Other symptoms also reported in the acute phase, such as dyspnea on exertion 94.2%, fever 88.5%, cough 85.7% and chills 85.7% were more frequent in the male population than in the female population (**Figure 1**). In the evaluation of persistent symptoms of long COVID, even after 4 months of hospital discharge, 91.3% (73/80) of patients reported having experienced one or more unexplained symptoms related to the acute infection. The most common symptoms reported in this period were myalgia (60%), fatigue (55%), headache (42.5%) and cough (35%). The female population manifested a higher proportion of almost all symptoms. Over the 2-year follow-up, 51/80 (63.7%) of patients still reported persistent long COVID symptoms. The most common symptoms in this period were fatigue (63.7%), hair loss (47.5%), arthralgia (42.5%), palpitation (38.7%), dyspnea on exertion (36.6%) and cough (33.8%). Participants had no reports of nausea, vomiting, history of fever, or dizziness. Women had a higher proportion of fatigue (66%) and hair loss (75.5%) than the male population, while men had a higher proportion of dyspnea on exertion (42.8%) and chest pain (28.5%).

### 3.3 Long COVID and vaccination status

**Figure 2:**
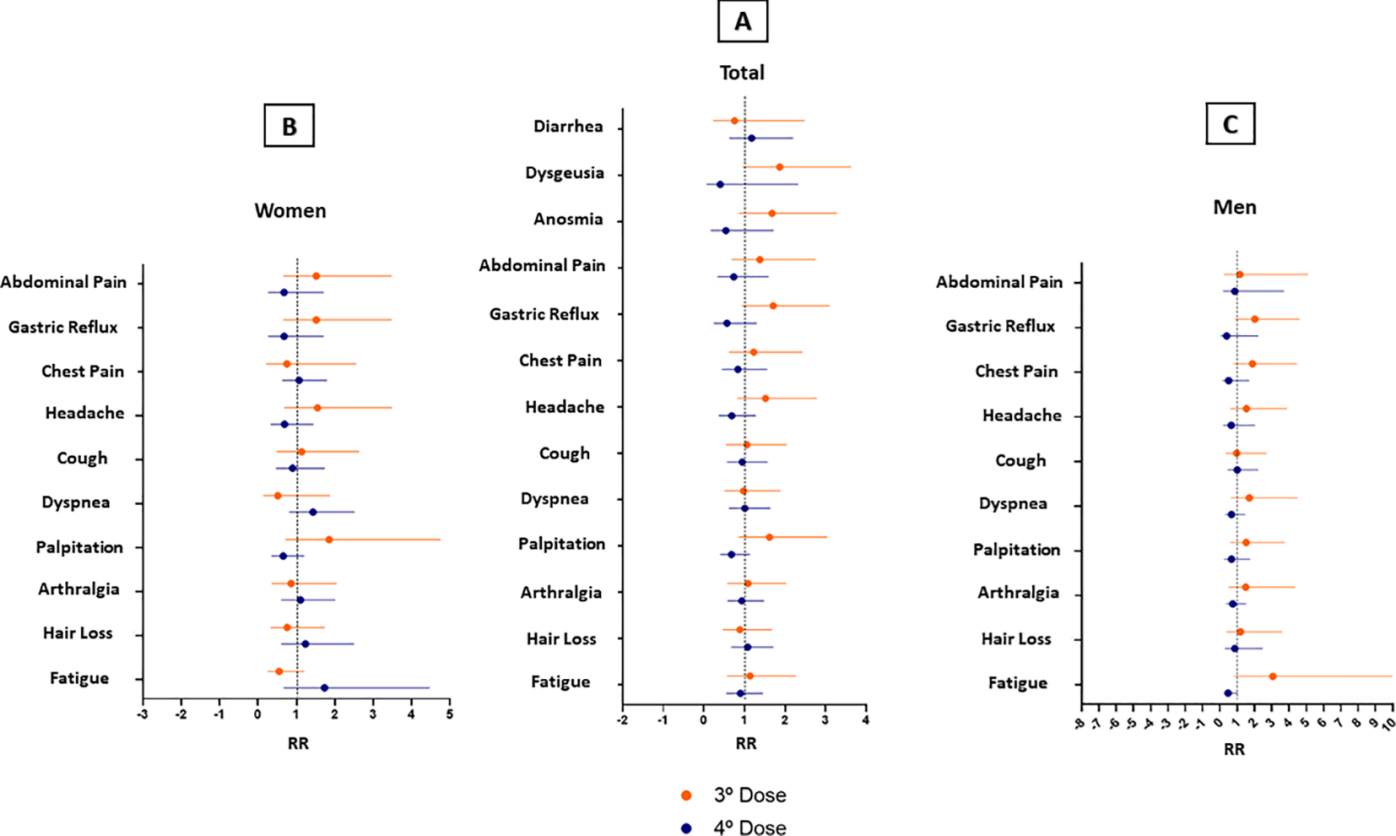
Long Covid symptoms in participants vaccinated in a 3-dose schedule versus a 4-dose schedule. **(A)** Total Patients **(B)** Women **(C)** Men. Error bars represent 95% CI.

We recorded the number and type of vaccines of SARS-CoV-2 administered until October 2022, when all participants had completed the 2-year follow-up assessments. The vaccination and booster were first applied in Amazonas to the elderly and health care professionals. In the study population, 92.5% (74/80) of participants were vaccinated with at least 1 dose of the SARS-CoV-2 vaccine. They had an average age of 52.8 years and had comorbidities such as diabetes and hypertension. Regarding the number of doses received by vaccinated participants, 2.5% (2/80) of patients took only the 1st dose; 22.5% (18/80) 2 doses, 18.8% (23/80) 3 doses, and 39.9% (31/80) 4 doses. The mean interval between the 1st and 2nd dose was 77 days ± 4.5 (95% CI: 68.6 - 86.8); and between the 1st and 4th dose was 438 days ± 9 (95% CI: 419.6 - 456.4) (**Table 3**). In our adjusted investigation, a four-dose regimen was associated with a greater protective effect and appeared to decrease the frequency of fatigue symptoms in the male population (**Figure 2**) (IRR 0.48, 95%CI: 0.22 - 1.02; *p=0.054*). When considering the remaining symptoms of long COVID, no significant differences in benefit were found between the dose regimens in females and the general population.

**Table 3:** Vaccination status of study participants after 2 years.

Vaccination coverage	N=80
Yes, n (%)	74/80 (92.5%)
**1° Dose, n (%)**	**74/80 (92.5%)**
Coronavac/(Sinovac)	17/80 (21.3%)
Pfizer/(BioNTech)	11/80 (13.8%)
Astrazeneca/(Oxford)	44/80 (55.0%)
Janssen	2/80 (2.5%)
**2° Dose, n (%)**	**72/80 (90%)**
Coronavac/(Sinovac)	17/80 (21.3%)
Pfizer/(BioNTech)	12/80 (15.0%)
Astrazeneca/(Oxford)	43/80 (53.8%)
**3° Dose, n (%)**	**54/80 (67.5%)**
Coronavac/(Sinovac)	44/80 (55.0%)
Pfizer/(BioNTech)	10/80 (12.5%)
**4° Dose, n (%)**	**31/80 (38.8%)**
Coronavac/(Sinovac)	1/80 (1.3%)
Pfizer/(BioNTech)	22/80 (27.5%)
Astrazeneca/(Oxford)	8/80 (10.0%)

### 3.4 Inflammatory profile and long COVID

**Figure 3:**
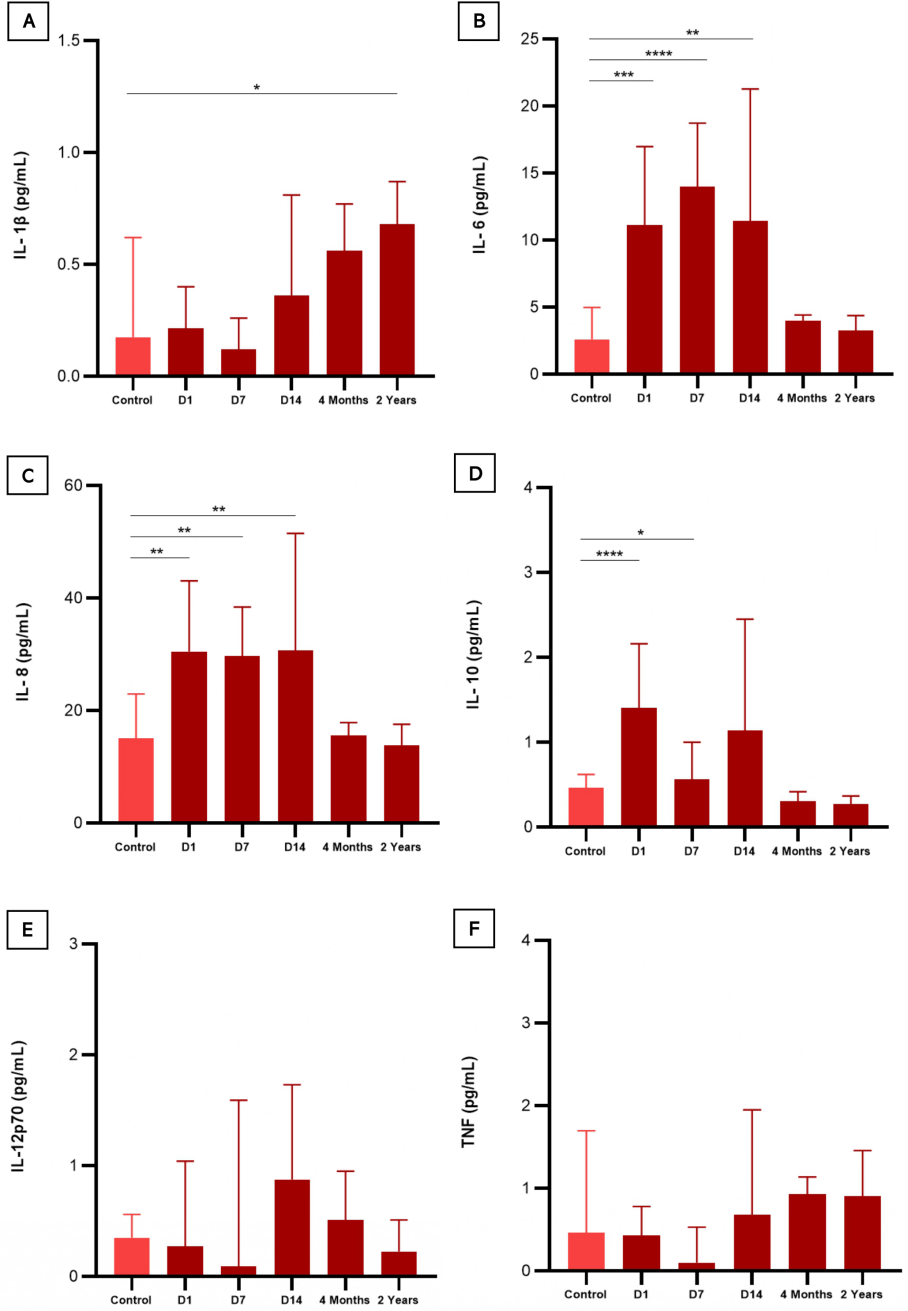
Serum levels of cytokines in COVID-19 infected patients compared to healthy participants (negative controls). **(A)** (IL-1β) interleukin 1-beta; **(B)** (IL-6) interleukin 6; **(C)** (IL-8) interleukin 8; **(D)** (IL-10) interleukin 10; **(E)** (IL-12p70) interleukin 12p70; and **(F)** (TNF) Tumor Necrosis Factor. Statistical difference was calculated by Mann-Whitney test. *p < 0,05, **p < 0,01, ***p < 0,001 e ****p < 0,0001.

**Figure 4:**
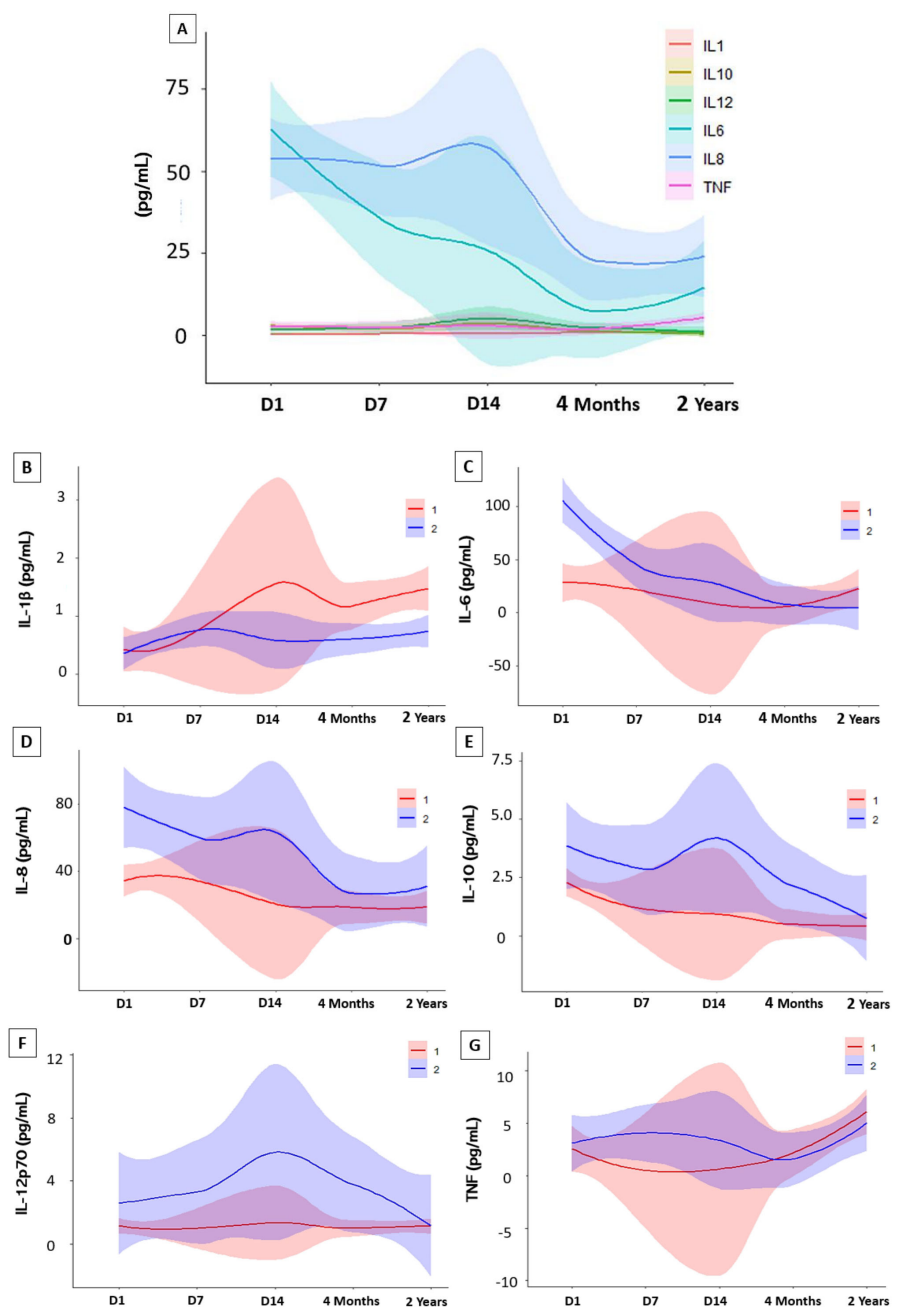
Variations in cytokine levels during follow-up. Statistical analysis were performed using interactions in a linear mixed-effects model. **(A)** Cytokine Trend Model of study participants in the Acute Phase (Day 1, Day 7 and Day 14) and Convalescent Phase (4 months and 2 years). Analysis of interactions between sex. **(B)** (IL-1β) interleukin 1-beta; **(C)** (IL-6) interleukin 6; **(D)** (IL-8) interleukin 8 **(E)** (IL-10) interleukin 10; **(F)** (IL-12p70) interleukin 12p70 and **(G)** (TNF) Tumor Necrosis Factor. Abbreviations: (1) Women; (2) Men.

When comparing the results of baseline cytokine values in controls versus follow-up times, as seen in **Figure 3**, four cytokines were significantly elevated. During the acute phase IL-6 (D1, D7 and D14), IL-8 (D1, D7 and D14) and IL-10 (D1 and D7) remained high compared to the control group, declining over time after the individual’s recovery, while IL-1β showed an inverse variation, remaining high after 2 years. A mixed linear regression model that explores the relationship between cytokine levels and different variables, including time (on different days after hospitalization), sex, and their interactions was also done (**Figure 4**). The **Figure 4** was created by combining the cytokines IL-1β, IL6, IL8, IL10, IL12p-70, and TNF, without distinction of sex over time. It is possible to see that the cytokines IL-1β, IL-6, and TNF intersect at some point in the graph, highlighting the proximity of this information at some period of the study, an inverse effect observed with IL-8, IL-10, and IL-12p70 despite the proximity of their confidence intervals. It is also possible to highlight the trend and variation of IL-6 and IL-8 among the other cytokines, which did not vary as much among themselves over time. To assess whether there was a trend of change in the levels of each cytokine (**Supplementary Table S1**), an estimation was performed for each time unit (Day 1, Day 7, Day 14) and (4 Months, 2 Years). Regarding IL-1β, the estimation coefficient at 4 months is 0.03 (95%CI: 0.00-0.06), which, on average, is expected to be an increase of 0.03 in units after 4 months, compared to day 1 of the acute phase. This effect is statistically significant at the 4-month and 2-year follow-up (*p=0.035* and *p<0.001*, respectively), indicating that IL-1β levels increase over time after acute infection, unlike IL-10, which had a negative coefficient of - 0.05 (95%CI: 0.08-0.02; *p=0.004*). To verify a possible influence of the participants’ sex among the different cytokines over time in **Figure 4B**, the figures draw a smoothed line that shows the trend of the data and a shadow that represents the 95% confidence interval. When observing these variations comparing the sexes, the analysis indicates an expected difference between the cytokine units between the sexes and a statistical effect, given that we obtained a significant result in IL-6 with a coefficient (0.12, *p<0.001*). The interactions between time and sex (D7, D14, 4 Months and 2 Years x Sex), explore whether the relationship between time and cytokine levels differs between the sexes after Day 1 of hospitalization. This variation is observed in both IL-6 and IL-1β, over the 2-year follow-up. Random effects include the residual variance (σ2) and the variance between subjects (τ00 id). The ICC (Intraclass Correlation Coefficient) indicates the variation in cytokine levels. The model includes the number of subjects (N id) and observations. The marginal R² indicates how much the variability in cytokine levels is explained by the model variables. The conditional R² indicates whether the variability is explained by the full model, including random effects (**Supplementary Table S1**).

**Figure 5:**
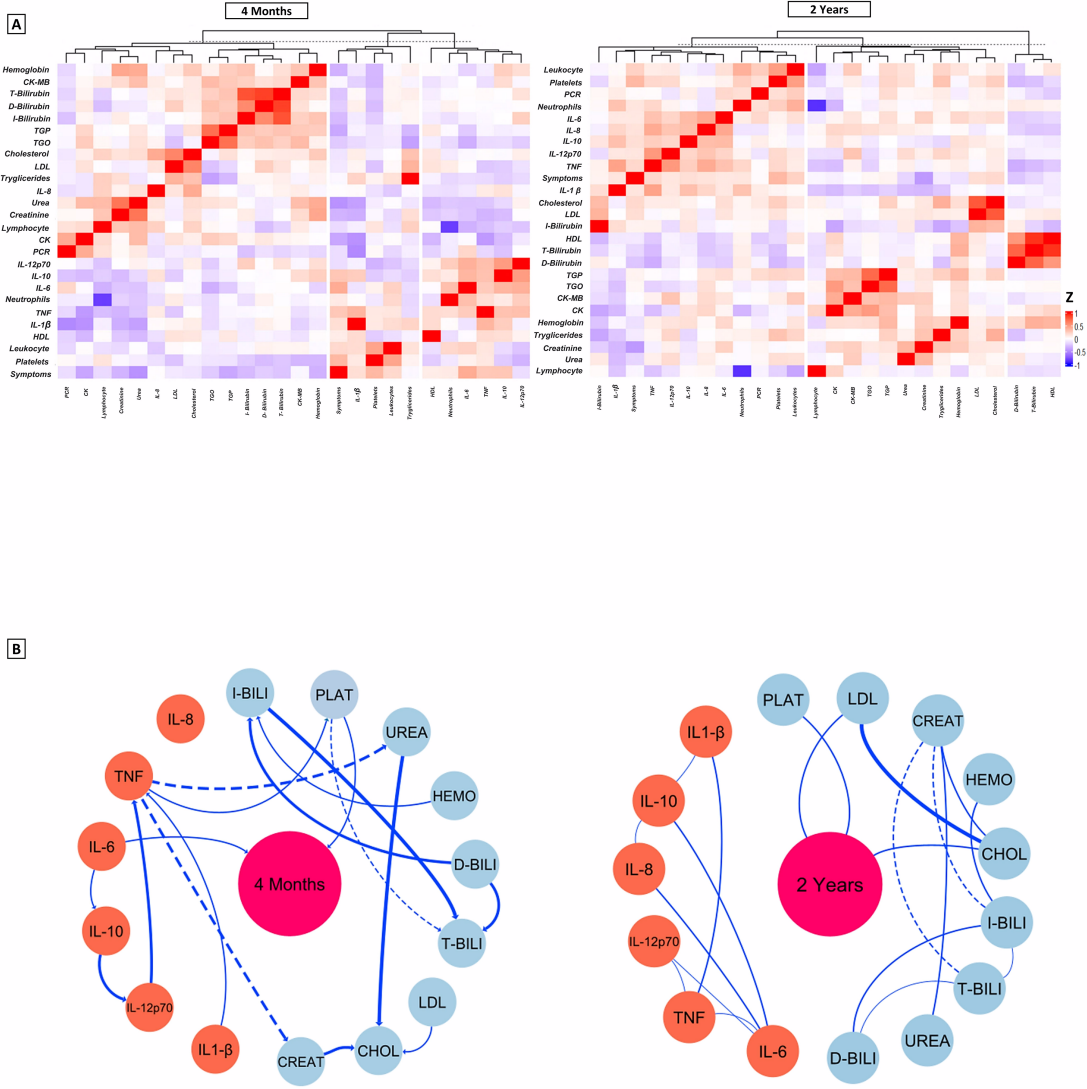
**(A)** Representative average heat map of the main functional and laboratory parameters at 4 months and 2 years. The settings configure the display of lines grouped by similarity based on Spearman Correlation, the colors represent the values of the variables in relation to the number of symptoms. The graphs emphasize the hierarchization of rows that group variables with similar patterns across observations, while the hierarchization of columns groups observations with similar patterns across observations. **(B)** Interaction networks between inflammatory and laboratory parameters and the number of Long Covid symptoms. Dashed lines between molecules show a negative correlation, while solid lines indicate a positive correlation. The thickness of these indicates the strength of the shine. The radiance index (r) used to categorize the strength of clarity as weak (r ≤ 0.35), moderate (r≥0.36 to r ≤ 0.67) or strong (r≥0.68). IQR, interquartile range; BMI, body mass index (calculated as weight in kilograms divided by height in meters squared) PLAT, Platelets; LDL, Low density lipoprotein; CREAT, Creatinine; HEMO, Hemoglobin; CHOL, Total Cholesterol; I-BILI, Indirect bilirubin; T-BILI, Total bilirubin; D-BILI, Direct bilirubin; IL-6, interleukin 6; IL-8, interleukin 8; TNF, Tumor Necrosis Factor; IL-12p70, interleukin 12p70; IL-10, interleukin 10; IL-1β, interleukin 1-beta.

In **Figure 5A**, we present the heatmaps that represent the profile of laboratory and inflammatory parameters after 4 months and 2 years. The heatmaps show the hierarchical clustering of these markers in individuals with persistent symptoms of long COVID. Patients had some significant interaction networks as shown in **Figure 5B**, when we investigated whether there was a correlation between cytokine values and laboratory parameters, with the number of long COVID symptoms. In the 4-month evaluation, patients showed a moderate positive correlation between Platelet values (R = 0.408, *p<0.001*), and negative for Urea (R = - 0.375, *p=0.002*). Regarding the cytokines evaluated at this time point, only IL-6 (R = 0.313, *p=0.011*) was positively correlated with the number of long COVID symptoms. After 2 years of evaluation, platelet values maintained a moderate positive correlation (R=0.398, p<0.001) together with Creatinine, with a negative correlation (R= - 0.410, *p<0.001*). These associations highlight the importance of these markers as possible indicators of the evolution of long COVID symptoms over time.

Regression analysis shows the influence of hematological, biochemical and inflammatory laboratory parameters on the multiplicative effect of the number of long COVID symptoms. In the analysis described in **Table 4** the cytokines IL-10 (IRR 1.770, *p=0.007*) and TNF, (IRR 1.025, *p=0.053*) were significantly and positively associated with the sum score of long COVID symptoms after 4 months of recovery. IL-12p70 was the cytokine most negatively associated with the number of symptoms. The other pro-inflammatory cytokines such as IL-1β and IL-6 were not associated with a multiplicative effect on symptoms. In the evaluation 2 years after hospital discharge, parameters such as Platelets and IL-12p70 were positively associated with a multiplier effect on symptoms, while Creatinine (IRR 0.387, *p=0.001*) was associated with a protective effect on the number of symptoms. The results highlight the importance of several laboratory and inflammatory parameters in the evolution of long COVID symptoms. They suggest that the inflammatory response and possible organ dysfunctions, such as renal and hepatic, may play a significant role in the persistence and severity of symptoms over time.

**Table 4:** Univariate and multivariate analysis of participants’ laboratory and inflammatory parameters, and their influence on the multiplicative probability of Long Covid symptoms at 4 months and 2 years after hospitalization.

4 Months	IRR	Univariable (*95%CI)*		*p-value*	IRR	Multivariable (*95%CI)*		*p-value*
IL-1β	1.112	0.981-1.260		0.095	0.956	0.848-1.078		0.472
IL-6	1.016	0.995	1.038	0.123				
IL-8	0.993	0.977	1.009	0.414				
IL-10	0.993	0.987	0.998	**0.014**	1.770	1.168	2.682	**0.007**
IL-12p70	0.994	0.990	0.998	**0.015**	0.734	0.587	0.917	**0.007**
TNF	1.045	1.014	1.077	**0.004**	1.025	0.999	1.052	**0.053**
Total bilirubin	0.588	0.238	1.450	0.249				
Direct bilirubin	0.975	0.209	4.546	0.974				
Indirect bilirubin	0.312	0.092	1.050	0.060	1.093	0.232	5.147	0.910
Hemoglobin	0.852	0.758	0.958	**0.008**	0.836	0.726	0.961	**0.012**
Leukocyte	1.000	0.999	1.000	0.359				
Neutrophil	1.004	0.986	1.023	0.603				
Lymphocyte	0.985	0.967	1.004	0.125				
Platelets	1.004	1.001	1.007	**0.006**	1.001	0.998	1.005	0.347
Creatinine	0.622	0.327	1.186	0.150				
Urea	0.999	0.999	0.999	**0.003**	0.999	0.999	0.999	**<0.001**
Alanine aminotransferase	0.990	0.976	1.004	0.184				
Aspartate aminotransferase	0.989	0.972	1.007	0.241				
CK	0.998	0.997	0.999	**0.009**	0.998	0.996	0.999	**0.002**
CK-MB	1.002	0.970	1.034	0.887				
C-reactive protein	1.017	0.998	1.036	0.066	1.020	0.992	1.050	0.159
LDL	1.000	0.995	1.005	0.812				
HDL	0.996	0.994	0.997	**<0.001**	1.000	0.994	1.006	0.786
Triglycerides	1.000	0.998	1.002	0.446				
Total cholesterol	1.001	0.998	1.004	0.317				
2 Years	IRR	(*95%CI)*	(*95%CI)*	*p-value*	IRR	(*95%CI)*	(*95%CI)*	*p-value*
IL-1β	1.023	0.995	1.052	0.103				
IL-6	0.992	0.983	1.001	0.094				
IL-8	0.999	0.997	1.002	0.779				
IL-10	0.928	0.824	1.045	0.221				
IL-12p70	1.065	1.005	1.130	**0.032**	1.058	1.023	1.094	**0.001**
TNF	1.007	1.000	1.014	**0.027**	1.000	0.996	1.005	0.680
Total bilirubin	0.853	0.644	1.130	0.268				
Direct bilirubin	0.315	0.090	1.100	0.070				
Indirect bilirubin	0.929	0.654	1.320	0.684				
Hemoglobin	0.999	0.910	1.096	0.984				
Leukocyte	1.000	1.000	1.000	**0.001**	1.000	0.999	1.000	0.301
Neutrophil	1.007	0.991	1.023	0.364				
Lymphocyte	0.995	0.979	1.012	0.616				
Platelets	1.000	1.000	1.000	**<0.001**	1.000	1.000	1.000	**0.047**
Creatinine	0.463	0.273	0.788	**0.005**	0.387	0.240	0.624	**<0.001**
Urea	0.987	0.973	1.001	0.072				
Alanine aminotransferase	1.001	0.995	1.007	0.578				
Aspartate aminotransferase	0.994	0.982	1.005	0.341				
CK	1.000	1.000	1.001	**0.013**	1.000	0.999	1.001	0.189
CK-MB	1.002	0.987	1.017	0.752				
C-reactive protein	1.002	0.999	1.000	0.058				
LDL	1.003	1.000	1.007	**0.031**	1.000	0.996	1.004	0.802
HDL	1.005	0.993	1.018	0.371				
Triglycerides	1.000	0.999	1.000	0.870				
Total cholesterol	1.002	1.000	1.005	**0.034**	1.002	0.999	1.005	0.127

IL-6, interleukin 6; (IL-8, interleukin 8; TNF, Tumor Necrosis Factor; IL-12p70, interleukin 12p70; IL-10, interleukin 10; IL-1β, interleukin 1-beta; CK, Creatine kinase; CKMB, Creatine kinase MB; LDL, Low density lipoprotein; HDL, High density lipoprotein; IRR, incidence rate ratio.Significant differences of p<0.05 are represented in bold.

## 4 Discussion

In this prospective cohort that followed up hospitalized individuals who survived acute COVID-19 in a pre-vaccination era, we sought to characterize and investigate the longitudinal relationship between inflammation markers and the development of long COVID. We found notable elevations in plasma inflammatory cytokine levels among individuals with long COVID. Furthermore, fatigue, dyspnea, fever and cough were the most common symptoms during the acute phase. Our findings show that even after recovery, a large number of long-term symptoms of musculoskeletal, cardiovascular and gastrointestinal origin still remained in the subjects even after 2 years, despite a considerable decline after 4 months, regardless of disease severity in the acute phase. These findings are similar to those found by Kim, et al., 2023 (19) who also found a high incidence of persistent symptoms of musculoskeletal and gastrointestinal origin. Overall, the prevalence of long COVID symptoms was 91.3% at 4 months and 63.7% at 2 years after diagnosis. This result is similar to that described by Geng, et al (20) in a cohort with 2,469 patients who reported the prevalence of long COVID in 65% of patients after 2 years. Few authors reported sex-disaggregated data, but overall, female patients were more likely to experience prolonged COVID-19 syndrome than their male counterparts, a trend observed in several studies (21). Other studies have reported that the risk of long-term symptoms is higher among previously hospitalized female patients than among males, with the female sex being a risk factor for chronic fatigue (22). These results suggest that the persistence of sequelae after SARS-CoV-2 infection is prolonged even among individuals with different acute disease profiles and has a greater impact on the female population, especially during the recovery phase. In addition to this gender difference in the influence of long COVID, several variants emerged after the first wave of the disease with different levels of transmission and virulence, such as the B.1.1.7 or Alpha SARS-CoV-2 variant, first isolated in the United Kingdom in September 2020. However, the impact of the variants on vaccination and long-term symptoms, as far as we know, was somewhat related compared to this association (23). Fernández-de-las-Peñas, et al. (24) showed that individuals infected with the historical Wuhan variant exhibited a greater number of post-COVID symptoms, than those patients infected with the Alpha or Delta variant. Therefore, it is important to consider the introduction of variants when determining the effect of symptoms in long COVID. Our study showed a small and significant protective effect of vaccination with 4 doses versus a 3-dose regimen in reducing long-term fatigue in men. Azzolini, et al. (25) found that the number of vaccine doses was associated with a lower prevalence of long COVID: 41.8% (95% CI, 37.0% - 46.7%) in unvaccinated patients and 16.0% (95% CI, 11.8% - 21.0%) with 3 doses. On the other hand, Wynberg, et al. (26) reported that most patients reported feeling that symptoms had not changed within a month after the first vaccination, regardless of the initial severity of COVID-19. Even with these new findings from studies reporting improvement in persistent symptoms after vaccination, the results are divergent. The inflammatory profile of our patients revealed higher levels of the inflammatory cytokines IL-1β and TNF after 2 years of hospital discharge, with the female population having a higher profile of IL-1β. The cytokines IL-1β, IL-10 and TNF showed a slight upward trend over the 4-month and 2-year follow-ups when compared with the baseline levels of the acute phase. Although IL-6 represents a key inflammatory factor in the acute phase, it did not show this same upward trend in the long term (27). The elevation of IL-1β and TNF may be associated with a possible acute pro-inflammatory reprogramming of long-lived lung macrophages or their precursors, which may result in a vicious cycle of production of these cytokines (28). Our regression also showed that TNF was positively associated with a multiplicative effect on the sum of long COVID symptoms at the 4-month follow-up. In a longitudinal analysis of the cytokine profile and its specific phenotype relationship in respiratory symptoms, it was shown that participants with persistent respiratory symptoms had significantly higher levels of IL-1β and TNF after 2 years. These results suggest that long COVID has evidence of being associated with residual inflammation, as reported by Moreno Perez, et al. (29). Patients with long COVID also present increased activation of the complement system. The complement system is a defense pathway against pathogens, including viruses, and triggers one or more activation pathways resulting from interaction with pathogens. Carlo Cervia-Hasler et al. (9) reported high levels of C2, C5bC6, Factor B molecules during severe acute disease and up to 6 months after recovery in a cohort with 39 healthy controls and 113 patients with COVID-19 for up to 1 year after initial confirmation of acute infection, and Afzali, et al., 2022. who notes that patients with severe covid have high levels of C5a and fragments of the C5b-9 terminals (7), these results indicate a possible activation of the classical or lectin pathway. Since complement activation involves hemolysis; induction of thromboinflammatory responses, including platelet and endothelial activation, an increase in serum levels of von Willebrand factor (vWF) and thrombospondin-1 (TSP-1) was also observed, in addition to high levels of MASP-2/C1Inh and C1s/C1Inh described by Lynch, et al. (8) which may explain the persistent inflammatory profile in this population. This study has some limitations, which should be considered. For instance, long COVID symptoms were self-reported by patients. It is possible that using different scales that assess different symptoms (fatigue or dyspnea) could reveal potential differences between groups. The lack of inclusion of uninfected controls, non-hospitalized or asymptomatic patients limits the ability to assess a direct association of SARS-CoV-2 infection with general and specific long COVID symptoms after 2 years. Our findings included patients discharged from the hospital in 2020 and therefore would not include those infected with other variants different from those circulating in Manaus, Brazil, by the time of acute infection.

## 5 Conclusions

Although the frequency of long COVID symptoms declines over time after the acute illness, symptoms persist for more than 2 years after hospital discharge. Vaccination with a fourth dose booster appears to significantly influence the male population in reducing symptoms of fatigue associated with long COVID. Furthermore, elevated serum levels of IL-1β, IL-10, and TNF, as well as low levels of IL-6 and IL-8, IL-12p70, appear to constitute a cytokine long COVID profile. In our study, we demonstrated that it was possible to identify important positive correlations between platelet counts and the number of long COVID symptoms, even after 2 years. As the components of the complete blood count are readily available, we recommend the routine use of this biomarker in the monitoring of long COVID. Therefore, we suggest continued research on long COVID to better understand the potential long-term health consequences of COVID-19, including symptom persistence, impact on quality of life and the efficacy of interventions such as vaccination.

## Data Availability

The raw data supporting the conclusions of this article will be made available by the authors, without undue reservation.
