# Supplementary material for: Inflammatory discoveries two years after acute severe COVID-19: a longitudinal biomarker profile assessment in long COVID individuals in the Brazilian Amazon
Source: Front Immunol. 2024 Dec 23;15:1520193. doi: 10.3389/fimmu.2024.1520193 (PMC11700803; doi:10.3389/fimmu.2024.1520193)
Supplement: Supplementary file 1 [file Table1.docx]

Supplementary Material

## Supplementary Tables

**Supplementary Table S1.** A mixed linear regression model that explores the relationship between cytokine levels and different variables including time (on different days after hospitalization), sex, and their interactions.

|  | **TNF** |  |  |  | **IL-12p70** |  |  |
| --- | --- | --- | --- | --- | --- | --- | --- |
| *Predictors* | *Estimates* | *95%IC* | *pvalue* | *Predictors* | *Estimates* | *95%IC* | *pvalue* |
| (Intercept) | (-0.01) | (-0.04 / 0.01) | 0.327 | (Intercept) | (-0.01) | (-0.00 / 0.02) | 0.502 |
| D7 | (-0.01) | (-0.06 / 0.03) | 0.593 | D7 | (-0.01) | (-0.04 / 0.02) | 0.642 |
| D14 | (-0.00) | (-0.12 / 0.12) | 0.973 | D14 | (0.01) | (-0.08 / 0.10) | 0.851 |
| 4 months | (-0.00) | (-0.04 / 0.04) | 0.991 | 4 months | (-0.00) | (-0.03 / 0.02) | 0.812 |
| 2 years | (0.06) | (0.02 / 0.09) | **0.001** | 2 years | (-0.00) | (-0.02 / 0.02) | 0.974 |
| Sex [2] | (0.01) | (-0.03 / 0.05) | 0.650 | Sex [2] | (0.02) | (-0.02 / 0.06) | 0.272 |
| D7 X Sex [2] | (0.02) | (-0.04 / 0.08) | 0.521 | D7 X Sex [2] | (-0.00) | (-0.05 / 0.04) | 0.866 |
| D14 X Sex [2] | (0.01) | (-0.13 / 0.14) | 0.926 | D14 X Sex [2] | (0.01) | (-0.09 / 0.10) | 0.877 |
| 4 months X Sex [2] | (-0.02) | (-0.07 / 0.03) | 0.470 | 4 months X Sex [2] | (0.01) | (-0.03 / 0.05) | 0.655 |
| 2 years X Sex [2] | (-0.04) | (-0.09 / 0.01) | 0.138 | 2 years X Sex [2] | (-0.02) | (-0.09 / 0.02) | 0.286 |
|  |  |  |  |  |  |  |  |
| **Random effects** |  |  |  | **Random effects** |  |  |  |
| σ2 | (0.01) |  |  | σ2 | (0.00) |  |  |
| τ00id | (0.00) |  |  | τ00id | (0.00) |  |  |
| ICC | (0.17) |  |  | ICC | (0.55) |  |  |
| N id | 80 |  |  | N id | 80 |  |  |
|  |  |  |  |  |  |  |  |
| Observations | 285 |  |  | Observations | 285 |  |  |
| Marginal R2 / Conditional R2 | (0.060)/(0.218) |  |  | Marginal R2 / Conditional R2 | (0.021)/(0.563) |  |  |
|  |  |  |  |  |  |  |  |
|  | **IL-10** |  |  |  | **IL-8** |  |  |
| *Predictors* | *Estimates* | *95%IC* | *pvalue* | *Predictors* | *Estimates* | *95%IC* | *pvalue* |
| (Intercept) | (0.02) | (-0.01 / 0.05) | 0.261 | (Intercept) | (-0.00) | (-0.02 / 0.01) | 0.609 |
| D7 | (-0.04) | (-0.08 / -0.00) | **0.043** | D7 | (0.00) | (-0.03 / 0.04) | 0.912 |
| D14 | (-0.03) | (-0.14 / 0.08) | 0.622 | D14 | (-0.00) | (-0.09 / 0.09) | 0.947 |
| 4 months | (-0.05) | (-0.08 / -0.02) | **0.004** | 4 months | (-0.00) | (-0.03 / 0.02) | 0.807 |
| 2 years | (-0.05) | (-0.08 / -0.02) | **0.002** | 2 years | (0.02) | (-0.00 / 0.05) | 0.065 |
| Sex [2] | (0.04) | (-0.00 / 0.08) | 0.063 | Sex [2] | (0.01) | (-0.02 / 0.04) | 0.490 |
| D7 X Sex [2] | (-0.01) | (-0.07 / 0.04) | 0.601 | D7 X Sex [2] | (-0.01) | (-0.05 / 0.04) | 0.727 |
| D14 X Sex [2] | (0.00) | (-0.12 / 0.12) | 0.975 | D14 X Sex [2] | (-0.00) | (-0.10 / 0.10) | 0.991 |
| 4 months X Sex [2] | (-0.01) | (-0.06 / 0.03) | 0.539 | 4 months X Sex [2] | (-0.01) | (-0.05 / 0.03) | 0.628 |
| 2 years X Sex [2] | (-0.03) | (-0.07 / 0.02) | 0.198 | 2 years X Sex [2] | (-0.03) | (-0.07 / 0.00) | 0.084 |
|  |  |  |  |  |  |  |  |
| **Random effects** |  |  |  | **Random effects** |  |  |  |
| σ2 | (0.01) |  |  | σ2 | (0.00) |  |  |
| τ00id | (0.00) |  |  | τ00id | (0.00) |  |  |
| ICC | (0.45) |  |  | ICC |  |  |  |
| N id | 80 |  |  | N id | 80 |  |  |
|  |  |  |  |  |  |  |  |
| Observations | 285 |  |  | Observations | 285 |  |  |
| Marginal R2 / Conditional R2 | (0.084)/(0.497) |  |  | Marginal R2 / Conditional R2 | (0.023)/NA |  |  |
|  |  |  |  |  |  |  |  |
|  | **IL-6** |  |  |  | **IL-1β** |  |  |
| *Predictors* | *Estimates* | *95%IC* | *pvalue* | *Predictors* | *Estimates* | *95%IC* | *pvalue* |
| (Intercept) | (-0.00) | (-0.03 / 0.03) | 0.952 | (Intercept) | (-0.02) | (-0.04 / 0.00) | 0.111 |
| D7 | (-0.01) | (-0.06 / 0.04) | 0.803 | D7 | (-0.01) | (-0.05 / 0.03) | 0.553 |
| D14 | (-0.03) | (-0.16 / 0.11) | 0.688 | D14 | (0.06) | (-0.05 / 0.16) | 0.270 |
| 4 months | (-0.04) | (-0.08 / 0.00) | 0.076 | 4 months | (0.03) | (0.00 / 0.06) | **0.035** |
| 2 years | (-0.01) | (-0.05 / 0.03) | 0.652 | 2 years | (0.07) | (0.04 / 0.10) | **<0.001** |
| Sex [2] | (0.12) | (0.08 / 0.16) | **<0.001** | Sex [2] | (-0.01) | (-0.04 / 0.03) | 0.743 |
| D7 X Sex [2] | (-0.10) | (-0.17 / -0.03) | **0.005** | D7 X Sex [2] | (0.04) | (-0.02 / 0.09) | 0.170 |
| D14 X Sex [2] | (-0.09) | (-0.24 / 0.05) | 0.208 | D14 X Sex [2] | (-0.05) | (-0.16 / 0.07) | 0.413 |
| 4 months X Sex [2] | (-0.12) | (-0.18 / -0.06) | **<0.001** | 4 months X Sex [2] | (-0.02) | (-0.07 / 0.03) | 0.401 |
| 2 years X Sex [2] | (-0.14) | (-0.20 / -0.09) | **<0.001** | 2 years X Sex [2] | (-0.05) | (-0.10 / -0.01) | **0.023** |
|  |  |  |  |  |  |  |  |
| **Random effects** |  |  |  | **Random effects** |  |  |  |
| σ2 | (0.01) |  |  | σ2 | (0.00) |  |  |
| τ00id | (0.00) |  |  | τ00id | (0.00) |  |  |
| ICC | (0.07) |  |  | ICC | (0.18) |  |  |
| N id | 80 |  |  | N id | 80 |  |  |
|  |  |  |  |  |  |  |  |
| Observations | 285 |  |  | Observations | 285 |  |  |
| Marginal R2 / Conditional R2 | (0.200)/(0.254) |  |  | Marginal R2 / Conditional R2 | (0.102)/(0.266) |  |  |
